# Protracted Impairment of Maternal Metabolic Health in Mouse Dams Following Pregnancy Exposure to a Mixture of Low Dose Endocrine-Disrupting Chemicals, a Pilot Study

**DOI:** 10.3390/toxics9120346

**Published:** 2021-12-09

**Authors:** Alyssa K. Merrill, Timothy Anderson, Katherine Conrad, Elena Marvin, Tamarra James-Todd, Deborah A. Cory-Slechta, Marissa Sobolewski

**Affiliations:** 1 Department of Environmental Medicine, University of Rochester School of Medicine, Rochester, NY 14642, USA; alyssa_merrill@urmc.rochester.edu (A.K.M.); velociteal@gmail.com (T.A.); bachmannkatherine@gmail.com (K.C.); elena_suk@urmc.rochester.edu (E.M.); Deborah_Cory-slechta@urmc.rochester.edu (D.A.C.-S.); 2 Department of Environmental Health, Harvard University, Boston, MA 02115, USA; tjtodd@hsph.harvard.edu

**Keywords:** type 2 diabetes, maternal health, pregnancy, endocrine-disrupting chemicals

## Abstract

Pregnancy, a period of increased metabolic demands coordinated by fluctuating steroid hormones, is an understudied critical window of disease susceptibility for later-life maternal metabolic health. Epidemiological studies have identified associations between exposures to various endocrine-disrupting chemicals (EDCs) with an increased risk for metabolic syndrome, obesity, and diabetes. Whether such adverse outcomes would be heightened by concurrent exposures to multiple EDCs during pregnancy, consistent with the reality that human exposures are to EDC mixtures, was examined in the current pilot study. Mouse dams were orally exposed to relatively low doses of four EDCs: (atrazine (10 mg/kg), bisphenol-A (50 µg/kg), perfluorooctanoic acid (0.1 mg/kg), 2,3,7,8-tetrachlorodibenzo-p-dioxin (0.036 µg/kg)), or the combination (MIX), from gestational day 7 until birth or for an equivalent 12 days in non-pregnant females. Glucose intolerance, serum lipids, weight, and visceral adiposity were assessed six months later. MIX-exposed dams exhibited hyperglycemia with a persistent elevation in blood glucose two hours after glucose administration in a glucose tolerance test, whereas no such effects were observed in MIX-exposed non-pregnant females. Correspondingly, MIX dams showed elevated serum low-density lipoprotein (LDL). There were no statistically significant differences in weight or visceral adipose; MIX dams showed an average visceral adipose volume to body volume ratio of 0.09, while the vehicle dams had an average ratio of 0.07. Collectively, these findings provide biological plausibility for the epidemiological associations observed between EDC exposures during pregnancy and subsequent maternal metabolic dyshomeostasis, and proof of concept data that highlight the importance of considering complex EDC mixtures based of off common health outcomes, e.g., for increased risk for later-life maternal metabolic effects following pregnancy.

## 1. Introduction

Historically, toxicological research has studied maternal exposures to understand the health of the fetus but has ignored the long-term health of the mother [1], despite the fact that pregnancy represents a unique critical window for later-life maternal metabolic health due to the unique metabolic demands of supporting fetal development [2,3]. Pregnancy-specific changes in maternal metabolic physiology, i.e., lower fasting glucose, increased insulin secretion, elevated adipose volume and decreased insulin sensitivity, occur synchronously with predictable patterns of endocrine alterations [4,5]. Given that pregnant women are exposed concurrently to multiple endocrine-disrupting chemicals (EDCs) [6,7,8,9], pregnancy-specific metabolic demands raise concern that EDC exposures may induce unique, protracted impairments of maternal metabolic health. Recent epidemiological studies have reported associations between EDC exposures, such as persistent organic pollutants, per and poly-fluoroalkyl substances, and bisphenols, during pregnancy and an increased risk of maternal hyperglycemia and gestational diabetes [10,11,12,13,14,15,16,17,18,19,20]. Following pregnancy, women undergo a metabolic reset to return to the physiological state experienced prior to pregnancy adaptations, the persistence of these pregnancy-specific changes in maternal metabolic physiology increases the risk of later-life maternal metabolic disease [21,22]. Furthermore, epidemiological studies have found per and poly-fluoroalkyl substances and phthalates exposure during pregnancy to be associated with increased postpartum maternal adiposity [23,24]. Pregnancy phthalate exposures have also been found in an epidemiological study to be associated with postpartum maternal elevated plasma glucose, insulin resistance, and adverse lipid changes, i.e., lower HDL and higher triglyceride levels [25]. Studies of non-pregnant women have also shown these same chemicals to be associated with obesity and type 2 diabetes [26,27,28]. Yet, little remains known about the protracted impact of EDC exposures during pregnancy on later-life maternal metabolic health, including type 2 diabetes risk. Understanding the influence of EDC exposures during pregnancy on later-life maternal metabolic health is critical, as worldwide, approximately 1 in 10 women are living with a form of diabetes [29], a quantity expected to double in the next decade [30].

Complicating the ability to predict metabolic disease risk, pregnant women are exposed to EDC mixtures, not to single EDCs [9,25,31,32,33,34]. If combinatorial EDCs confer an enhanced effect on metabolic health, risk assessment based on no adverse effect levels for single EDC exposures could overestimate “safe” exposure levels. Multiple studies focused on reproductive toxicity support this possibility, as the reproductive toxicity of EDC mixtures indicates dose-additive effects of low dose mixtures [35,36,37,38], even when single EDC exposures show no phenotype [39,40,41]. This study hypothesizes that well-studied EDCs known to target metabolic function through different direct molecular targets will produce enhanced metabolic toxicity on a single physiological function, such as glucose metabolism [35,42]. Glucose homeostasis requires the proper functioning of multiple interacting physiological systems [43,44]. Our multiple hits hypothesis [42] predicts that low-dose EDC hits at multiple molecular target sites may overwhelm compensatory mechanisms that normally maintain metabolic homeostasis [28,45], thereby increasing metabolic disease risk. Given the increased metabolic demands of pregnancy and the rapid metabolic changes that occur postpartum, it is essential to study EDC mixtures in pregnant and non-pregnant animals to determine if pregnancy alters sensitivity to EDC exposures with later-life metabolic consequences. This approach is critical to ensure that no adverse effect levels set for single EDC exposures are not underestimated.

Single chemical studies have identified approximately a dozen EDCs that have strong and substantial evidence of metabolic disruption [27,28]. Four well-studied EDC classes—herbicides, plasticizers, perfluorinated compounds, and PCB-like dioxin compounds—target glucose tolerance, lipid metabolism, and weight gain, although typically these studies have focused on male rodents [26,27,28,46,47]. Atrazine, a commonly used herbicide, modifies insulin resistance [48], and alters free fatty acids and hepatic lipids [49]. Bisphenol-A (BPA), a plasticizer, leads to hyperglycemia [27,50,51,52,53,54,55,56], increases body and adipose weight [57,58], and alters adipocyte hypertrophy [59] and differentiation [60]. Perfluorooctanoic acid, a perfluorinated compound [61,62], induces hypercholesterolemia, and increases liver weight, bodyweight, adipokines, and lipids [63,64,65,66]. 2,3,7,8-tetrachlorodibenzo-p-dioxin [67,68], a PCB-like dioxin compound, has been shown to lower insulin secretion [69,70,71] and decrease serum lipids [72]. With the exception of BPA [51,52], most animal models have not determined the potential protracted effects of EDC exposures, either singly or in mixtures, on pregnant females on later-life maternal metabolic health.

To address these gaps, the current pilot study assessed the effects of pregnancy exposures to low-dose EDCs either singly or in a mixture on later-life maternal metabolic health. Pregnant mice (hereafter referred to as dams) were exposed to low doses of four EDCs singly shown to have high to moderate evidence for metabolic disruption (atrazine, bisphenol-A, perfluorooctanoic acid, and 2,3,7,8-tetrachlorodibenzo-p-dioxin) or to a mixture (MIX) of these four from gestational day 7 until birth [27]. Additionally, non-pregnant female mice were exposed to MIX for an equivalent 12 days to determine the role of pregnancy. Approximately 6 months after the cessation of exposure, dams and non-pregnant females underwent a glucose tolerance test, and serum lipids, visceral adiposity, and serum adipokines were measured.

## 2. Materials and Methods

### 2.1. Animals and Husbandry

To determine whether EDC exposures occurring singly or in a mixture alter later-life maternal metabolic disease risk, adult (postnatal day (PND) 60) male and female C57BL/6J mice obtained from Jackson Laboratories (Bar Harbor, ME, USA) were bred using a monogamous pairing scheme. Male and female mice were paired, and the following morning, female mice were checked for the presence of a mucus plug, which was denoted as gestational day (GD) 0. If a mucus plug was observed, males were removed from the home cage. Pregnant dams remained singly housed throughout weaning. Pregnant dams were exposed orally via EDC-spiked mealworm to either a single EDC at a low dose or the combination of all four EDCs at the same doses, as given in the single exposures: atrazine (ATR—10 mg/kg), bisphenol-A (BPA—50 µg/kg), perfluorooctanoic acid (PFOA—0.1 mg/kg), 2,3,7,8-tetrachlorodibenzo-p-dioxin (TCDD—0.036 µg/kg), or their mixture (MIX), from GD 7 until birth (*n* = 4/group for the weekly weights, glucose tolerance test, serum lipids, serum adipokines, and serum corticosterone, and *n* = 7/group for the visceral adipose visualization performed sequentially). Single EDC doses were considered relatively low because they were either: at or below levels typically shown to produce effects in animal studies (ATR [73,74,75,76], PFOA [65,77,78], and TCDD [69,70,72,79]), at current oral human reference dose levels (BPA) (https://www.epa.gov/iris, accessed on 1 September 2021), or at or below the EPA’s NOAEL for animal studies (PFOA) [80] (Table 1. EDC Dosing Method). ATR (>98% pure) and BPA (99% pure) (Sigma-Aldrich, St. Louis, MO, USA) were each separately dissolved in peanut oil, PFOA (96% pure, Sigma-Aldrich, St. Louis, MO, USA) was dissolved in water, and TCDD (98% pure, Cambridge Isotopes, Cambridge, MA, USA) was dissolved in anisole and diluted in peanut oil. Calculated doses were injected into mealworm(s). MIX-mealworms were created by injecting BPA and PFOA and ATR and TCDD into two worms, respectively. Upon injection, the mealworms were immediately flash-frozen to prevent degradation and leakage. Dams were dosed via EDC-spiked mealworm(s) at the same time every day from GD 7 until birth, with vehicle dams given vehicle oil-spiked mealworms [81,82]. This dosing method was selected to eliminate bolus increases in blood sugar from sugar-based treats and to eliminate the influence of stress from gavage administration. Furthermore, we dosed daily to ensure each dam had an equivalent behavioral experience across pregnancy. The limitation of daily dosing is that each EDC has a different half-life influencing bioaccumulation. Pregnancies were monitored daily to evaluate differences in time to parturition, litter size, or sex ratio of the offspring. On average, litters contained 7.19 ± 1.55 pups. Upon weaning at PND 25, dams remained individually housed in a vivarium room maintained at 22 ± 2 °C with a 12-h light–dark cycle (lights on at 06:00 h) for 18 weeks with weight measured weekly until metabolic reprogramming was assessed. Labdiet Rodent 5001 diet was provided ad libitum throughout the entire experimental duration.

To determine whether any effects of the mixture of EDCs six months postpartum were uniquely produced by pregnancy, sequentially a separate cohort of non-pregnant female mice underwent acute MIX exposure under the same experimental conditions and concurrently with the MIX dams for visceral adipose visualization. Adult (PND60) female C57BL/6J mice were obtained from Jackson Laboratories (Bar Harbor, ME). The female mice were singly housed beginning on day one. Oral exposure to either MIX or a vehicle oil-spiked mealworm started on day seven (*n* = 5/group). Exposure occurred for approximately twelve days to mimic the average length of exposure pregnant dams received. Following the cessation of exposure, non-pregnant female mice remained individually housed in a vivarium room maintained at 22 ± 2 °C with a 12-h light–dark cycle (lights on at 06:00 h) for 21 weeks with bodyweight measured weekly, starting three weeks after the cessation of exposure to duplicate the weekly bodyweight measurements in pregnant exposed dams until metabolic reprogramming was assessed. Labdiet Rodent 5001 diet was provided ad libitum throughout the entire experimental duration.

All experiments were carried out according to NIH Guidelines and were approved by the University of Rochester Medical School University Committee on Animal Resources (UCAR-2018-025-102139, 19 December 2018), which included a hazard plan with hazard notification signage to ensure proper handling of cages and bedding.

### 2.2. Glucose Tolerance Test

Six months after the cessation of exposure, an intraperitoneal glucose tolerance test was performed independent of estrous cyclicity [83]. Mice were transferred to cages with food removed while retaining water at 02:00 h. Fasting occurred for 6 h prior to testing at 08:00 h. Weights were obtained at the end of the fasting duration. A tail snip was performed once fasting commenced providing blood for glucose measurements. Baseline glucose was measured immediately following the tail snip utilizing an AlphaTRAK Blood Glucose Monitoring System (Zoetis, Parsippany, NJ, USA) on the setting for dogs. Each mouse was then intraperitoneally injected with a freshly prepared solution of glucose (Sigma-Aldrich, St. Louis, MO, USA) at 2 g/kg, after which blood glucose measurements were obtained using the AlphaTRAK glucometer at 15, 30, 60, and 120 min. After the final glucose measurement, mice were immediately sacrificed by live decapitation with trunk blood collected.

### 2.3. Serum Lipids

In pregnant exposed dams, triglycerides were determined in duplicate utilizing fresh serum obtained following the glucose tolerance test using a colorimetric assay kit (Abcam, Cambridge, UK) carried out according to the manufacturer’s instructions with a dilution of 1:10. High-density lipoprotein (HDL) and low-density lipoprotein (LDL) were measured with a colorimetric assay kit (Abcam, Cambridge, UK) in duplicate according to the manufacturer’s directions, with the HDL run at a dilution of 1:50 and LDL run without dilution. Assays were run on a SynergyH1 Hybrid Reader (BioTek, Winooski, VT, USA) with Gen5 2.01 software. Sample replicates with coefficients of variation (CVs) greater than 15% were excluded from the analysis.

### 2.4. Visceral Adipose Visualization

Visualization of visceral adipose in pregnant exposed dams was performed six months postpartum on a Scanco Medical VivaCT 40 cone-beam-computed tomography (CT) (Scanco Medical, Brüttisellen, Switzerland). Dams were anesthetized utilizing continuous isoflurane gas during the scan, with one dam from each group initially euthanized by an intraperitoneal injection of Euthasol (Virbac, Fort Worth, TX, USA) to validate and develop the CT parameters. Throughout the scan, respiration rate and movement were monitored to ensure proper isoflurane administration. The scans were run on the following parameters: tube diameter 35.8 mm OD, resolution 35.0-micron isotropic voxels, 1048 samples, 500 projections over 180°, 200 ms integration time, and an energy of 45 kVp with the intensity/current at 177 micro-amps. Analysis was conducted with Scanco evaluation software V6.5 (Scanco Medical, Brüttisellen, Switzerland). Gaussian filters were utilized to reduce beam hardening, Gauss ∑0.8 and Gauss support 1 pixel. In all images, the scaling maximum was set to 10,000 to provide a clearer contrast between the visceral adipose and other soft tissue. The region of interest extended from below the lungs to the proximal end of the tail. Manual contours were drawn to exclude the visible layers of subcutaneous adipose and the femurs, which were tightly pressed against the abdomen, as well as to include the visceral adipose. Renditions were generated on Scanco’s Image Processing Language (IPL) and 3-dimensional viewer via a transparent concatenation to merge adipose and bone with the adipose displayed slightly transparent and colored with the ventral side as the front. Volumetric quantifications of the adipose were calculated through the utilization of Scanco’s “per mille” value of 40 (µ linear attenuation coefficient = 0.32 cm^−1^) to remove potential air and an upper threshold of 75 (µ = 0.600 cm^−1^) to remove bone and soft tissue.

### 2.5. Serum Adipokines

Adipokines were measured in the pregnant exposed dams’ serum obtained after the glucose tolerance test. Adiponectin was assessed in duplicate utilizing a Mouse Adiponectin ELISA kit (Invitrogen, Carlsbad, CA, USA) according to the manufacturer’s directions and analyzed on a SynergyH1 Hybrid Reader (BioTek, Winooski, VT, USA) with Gen5 2.01 software. Insulin, monocyte chemoattractant protein 1 (MCP-1), plasminogen activator inhibitor-1 (PAI-1 Total), and resistin were measured on a Mouse Adipokine Magnetic Bead Panel (Sigma-Aldrich, St. Louis, MO, USA) according to directions provided by the manufacturer and run on a Bio-Plex 200 (Bio-Rad, Hercules, CA, USA). Sample replicates with CVs higher than 15% were excluded from the analysis.

### 2.6. Serum Corticosterone

Serum corticosterone was measured in duplicate using commercially available enzyme immunoassay kits (Arbor Assays, Ann Arbor, MI, USA), according to the manufacturer’s specifications and run on a SynergyH1 Hybrid Reader (BioTek, Winooski, VT, USA) with Gen5 2.01 software. Sample replicates with CVs higher than 15% were excluded from the analysis. Standard curve CVs fell below 10%.

### 2.7. Statistical Analyses

Shapiro–Wilk’s test and the Bartlett test were used to assess the normality and homogeneity of variances, respectively. For the glucose tolerance test of pregnant dams, repeated-measure ANOVAs were conducted with treatment groups (ATR, BPA, PFOA, TCDD, MIX, and vehicle) with glucose area under the curve and glucose concentrations at the final two-hour draw compared using one-way ANOVA, with subsequent post-hoc tests (Student’s *t*-test). The glucose tolerance test of non-pregnant female mice was analyzed separately utilizing a repeated-measures ANOVA, and the glucose area under the curve and final glucose concentration were analyzed with a Student’s *t*-test. Serum lipids, adipokines, and corticosterone were analyzed with one-way ANOVA across all treatments (single EDCs, MIX, and control) with subsequent post-hoc tests (Student’s *t*-test). Visceral adipose was only measured in the control and MIX exposed dams; as such, only Student’s one-way *t*-tests were conducted. The effect of treatment groups on weekly weight gain was assessed utilizing a repeated-measures ANOVA. Outliers were removed following a statistically significant Grubb’s test (Graphpad Software Inc., San Diego, CA, USA). Out of 1070 data points, 9 outliers were removed. The outliers removed are as follows: serum lipids, 5 data points out of 96; serum adipokines, 4 data points out of 120. Triglycerides had 4 data points out of 24 removed due to technical error. No outliers were removed from the weekly weights, the glucose tolerance test, or the visceral adipose visualization. Statistical analyses were conducted using JMP Pro 14.0 (SAS Institute Inc., Cary, NC, USA). *p*-values ≤ 0.05 were considered statistically significant.

## 3. Results

### 3.1. Glucose Tolerance Test

**Figure 1:**
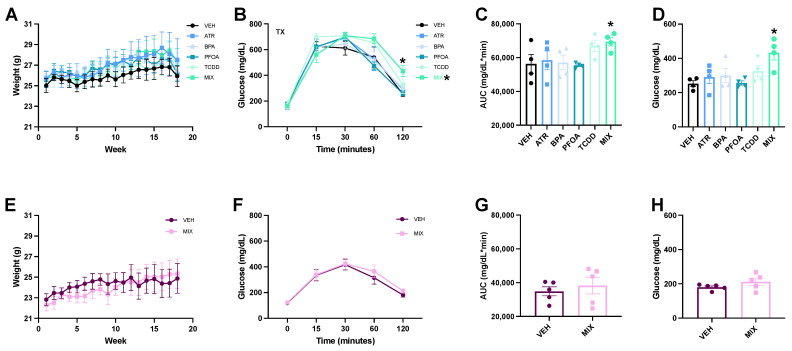
Weekly Weights and Glucose Tolerance Tests. (**A**) Dams were weighed weekly following weaning until the glucose tolerance test with no change in weight between groups at any week. Twenty-one weeks following the cessation of EDC exposures a glucose tolerance test was performed. (**B**) A marginally significant interaction was present when performing a repeated-measures ANOVA on the glucose concentrations over time in MIX dams with a significant effect of MIX at 120 min. (**C**) Only in the MIX dams was there a significantly elevated glucose area under the curve. (**D)** MIX dams also had significantly elevated glucose at the completion of the test; full data points displayed. (**E**) Non-pregnant female mice were weighed weekly three weeks after the cessation of exposure to mirror dams with no difference in weight at any week. (**F**) Non-pregnant MIX dams did not have a difference in blood glucose at any time point of the glucose tolerance test. (**G**) The non-pregnant MIX female mice did not have any difference in glucose area under the curve. (**H**) Non-pregnant MIX female mice did not differ in blood glucose at 120 min after the glucose injection. Data are reported as mean ± standard error (*n* = 4–5/group). Asterisk indicates *p* ≤ 0.05.

No significant effects of treatment on bodyweight were observed in pregnant exposed dams (F(5,16) = 0.22, *p* = 0.95, Figure 1A). Fasting blood glucose levels did not significantly differ between any treatment groups (F(5,18) = 1.0, *p* = 0.41, Figure 1B). Over the course of the glucose tolerance test, blood glucose concentrations exhibited a marginally significant interaction between time and treatment (F(20,72) = 1.5, *p* = 0.09), with MIX dams showing a significantly elevated glucose area under the curve compared to vehicle dams (*p* = 0.033, Cohen’s d = 1.63, Figure 1C). There were no significant differences in area under the curve in any other treatment group. Additionally, at the completion of the glucose tolerance test, 120 min following the glucose injection, only MIX dams exhibited significant hyperglycemia compared to vehicle dams (*p* = 0.001, Cohen’s d = 2.75, Figure 1D). Final blood glucose concentrations did not significantly differ in any other treatment group.

Non-pregnant MIX female mice, likewise, exhibited no bodyweight effects of treatment difference compared to non-pregnant vehicle female mice (F(1,8) = 0.060, *p* = 0.81, Figure 1E). As with the MIX dams, fasting blood glucose levels were not affected in the non-pregnant MIX female mice (t = −0.48, *p* = 0.64). Additionally, no difference was found in blood glucose levels in non-pregnant MIX female mice compared to non-pregnant vehicle female mice at any time point following the injection of glucose during the glucose tolerance test (Figure 1F). Nor was a significant interaction between time and treatment present (F(4,5) = 0.43, *p* = 0.71). Unlike the MIX dams, the non-pregnant MIX female mice did not have a significantly elevated glucose area under the curve (t = −0.62, *p* = 0.55, Figure 1G), nor did they show significantly elevated glucose at 120 min following the glucose injection (t = −1.44, *p* = 0.19, Figure 1H).

### 3.2. Serum Lipids

**Figure 2:**
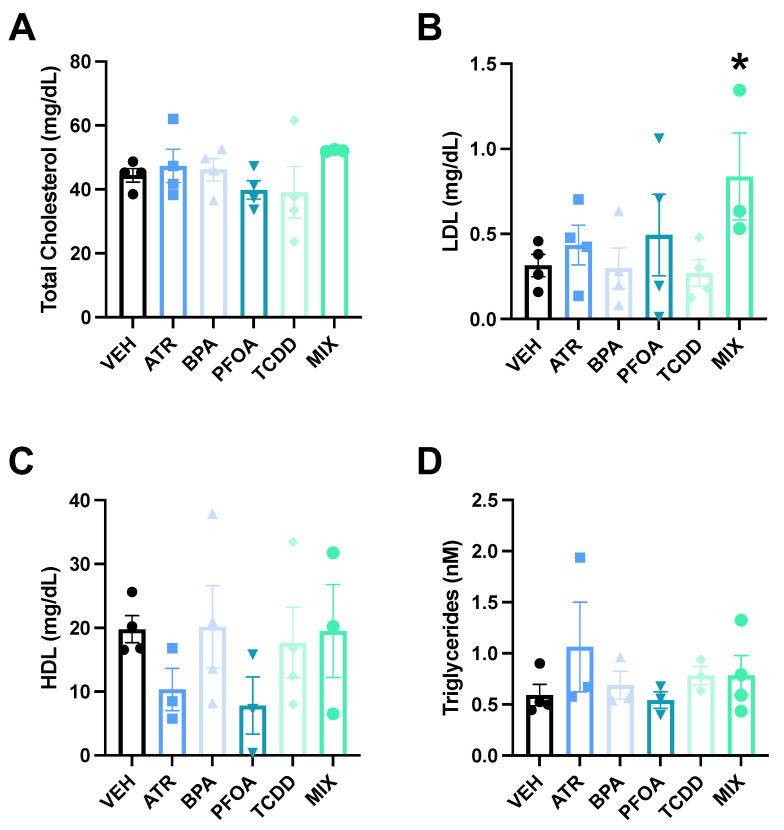
Pregnant Dams Serum Lipids. Following a glucose tolerance test, a cholesterol panel was performed on the serum. LDL (**B**) was significantly elevated only in the MIX dams. Total cholesterol (**A**), HDL (**C**), and triglycerides (**D**) did not differ in any treatment group. Data are presented as mean ± standard error (*n* = 3–4/group). Asterisk indicates *p* ≤ 0.05.

Compared to vehicle dams, MIX dams showed significant elevations in LDL (*p* = 0.037, Cohen’s d = 1.73, Figure 2B). There were no significant differences in LDL in any other treatment group. No differences in total cholesterol (F(5,17) = 1.02, *p* = 0.44), the HDL (F(5,15) = 0.98, *p* = 0.46) or the triglycerides (F(5,14) = 0.78, *p* = 0.58) were observed in any treatment group (Figure 2A,C,D).

### 3.3. Visceral Adipose Visualization

**Figure 3:**
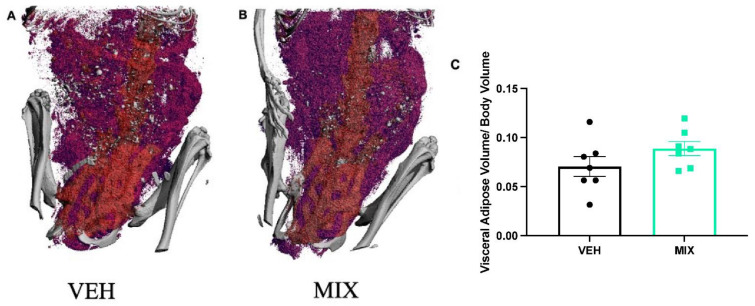
Pregnant Dams Visceral Adipose Visualization. Six months following the cessation of exposure, MIX and vehicle dams underwent a CT scan for visceral adipose assessment. (**A**) A representative image of the vehicle dams. (**B**) A representative image of the MIX dams. (**C**) MIX dams had a non-significant ratio of visceral adipose volume to body volume compared to vehicle dams. Data are shown as mean ± standard error (*n* = 7/group, *p* = 0.08).

No difference was observed in body volume, with a mean body volume of 7055 mm^3^ in vehicle dams and 7546 mm^3^ in MIX dams (t = −1.36, *p* = 0.10). MIX dams had a mean adipose volume of 673 mm^3^ in MIX dams compared to 511 mm^3^ in vehicle dams, though this failed to reach statistical significance (t = −1.57, *p* = 0.07, Cohen’s d = 0.83; data not shown). Furthermore, MIX dams had an average visceral adipose volume to body volume ratio of 0.09 while the vehicle dams had an average ratio of 0.07; however, this did not reach statistical significance (t = −1.49, *p* = 0.08, Cohen’s d = 0.79, Figure 3C).

### 3.4. Serum Adipokines

**Figure 4:**
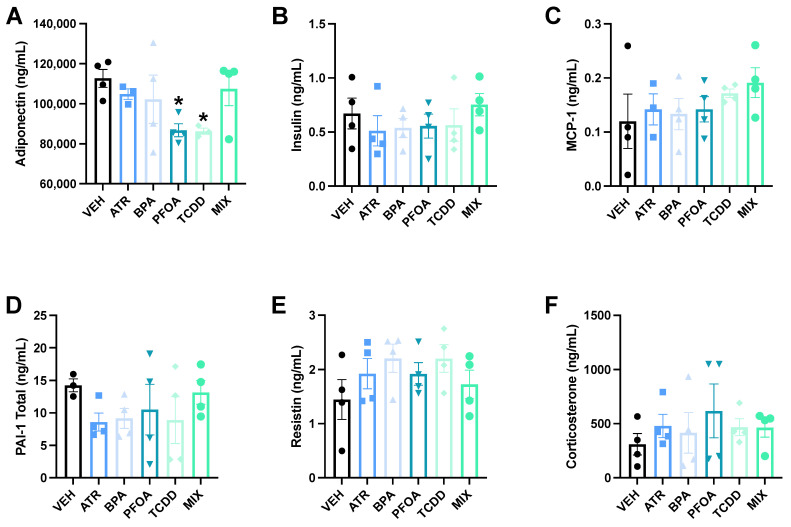
Pregnant Dams Serum Adipokines and Corticosterone. Various adipokines were analyzed following the glucose tolerance test: adiponectin (**A**), insulin (**B**), MCP-1 (**C**), PAI-1 total (**D**), and resistin (**E**). Adiponectin was significantly decreased in the single EDCs, PFOA and TCDD (**A**). No difference was observed between the remaining serum adipokines in any other treatment group. No difference was observed in corticosterone following the glucose tolerance test either (**F**). Data are shown as mean ± standard error (*n* = 3–4/group). Asterisk indicates *p* ≤ 0.05.

Adiponectin was significantly decreased by both PFOA (*p* = 0.017, Cohen’s d = 2.52) and TCDD (*p* = 0.023, Cohen’s d = 2.67) administered singly (Figure 4A). There were no significant changes in adiponectin in the other single EDC groups or in MIX dams. No differences in insulin (F(5,18) = 0.56, *p* = 0.73), MCP-1 (F(5,16) = 0.62, *p* = 0.69), PAI-1 Total (F(5,18) = 0.42, *p* = 0.83), or resistin (F(5,18) = 1.11, *p* = 0.39) were observed in any treatment group (Figure 4B–E).

### 3.5. Serum Corticosterone

Corticosterone levels at the completion of the glucose tolerance test did not differ between any treatment group (F(5,18) = 1.2, *p* = 0.34) (Figure 4F).

## 4. Discussion

Pregnancy is an understudied window of vulnerability for later-life maternal health [1,84], as the metabolic demands necessary for supporting fetal development may enhance maternal metabolic disease risk [2,3,85,86]. Recent epidemiological studies have shown an increased prevalence of life-long maternal metabolic alterations following gestational EDC exposures [23,24,25], yet few rodent models have explored the role of pregnancy per se by directly comparing non-pregnant and pregnant female mice, nor have they examined exposure to EDC mixtures, a phenomenon consistent with the human environment. The present pilot study examined the potential for protracted maternal metabolic toxicity following gestational EDC exposures, singly and in the mixture. In this pilot study, MIX exposure, but not single EDCs, produced hyperglycemia in MIX dams but not in non-pregnant MIX female mice. MIX-exposed dams showed significantly elevated blood glucose two hours after glucose administration. Furthermore, in preliminary analyses, MIX dams had elevated serum LDL, suggesting dyslipidemia. Despite higher means, there were no statistically significant increases in weight or visceral adipose. This study was a proof-of-concept experiment focused on a disease-centered approach to mixture studies aimed at investigating how EDCs with diverse molecular targets integrate across multiple signaling pathways in a complex system to evaluate joint effects on later-life maternal metabolic health [87,88,89]. More work is needed to replicate this study with a larger sample size and to investigate how additional EDC mixtures with diverse molecular modes of action may converge downstream to alter maternal health during gestation and across the lifespan.

Six months following the cessation of exposure, only MIX dams exhibited hyperglycemia during a glucose tolerance test, a phenotype often observed in metabolic syndrome and type 2 diabetes [90]. Furthermore, MIX dams had nearly double the blood glucose levels of vehicle dams at the 120 min time point of the glucose tolerance test. The stark differences in elevated total blood glucose (increased area under the curve values) and final blood glucose concentrations occurred without selection of a genetically predisposed strain and without a high-fat diet [44,48,91,92,93]. Interestingly, MIX dams did not show significant elevations in bodyweight. Although prior studies have shown that single EDC exposures are associated with hyperglycemia [50,51,52,53,54], such effects were not seen here in response to single EDC exposures. We hypothesize that variations in exposure concentration, exposure window, route of exposure, the time point of testing, and sex differences influence the effect of single EDC exposures on hyperglycemia. Furthermore, dependent on effect sizes, we may have been limited in identifying the effects of single EDCs. In an elegant series of studies, 10 and 100 µg/kg of BPA exposure via subcutaneous injection during pregnancy were shown to produce hyperglycemia as soon as five and four months postpartum, respectively, in OF-1 dams [51]. In our study, dams were exposed to 50 ug/kg BPA via oral exposure, which may alter toxicokinetic distribution. Alternatively, there may be mouse-strain-specific effects. Interestingly, single EDC exposures did produce multiple biochemical changes. For example, PFOA and TCDD alone resulted in reductions in adiponectin, indicating that these exposure concentrations were not “no adverse effect levels”, although these concentrations did not result in an overall increase in hyperglycemia at the completion of the glucose tolerance test. Importantly, MIX effects on blood glucose concentrations were unique to pregnant dams, as females exposed for an equivalent number of days showed no significant elevations in blood glucose six months later.

In addition to hyperglycemia, only MIX dams showed elevated serum LDL. Dyslipidemia is another phenotype commonly observed in individuals with metabolic dysfunction, such as insulin resistance and type 2 diabetes, and has been utilized as a predictor for these diseases [43,94,95,96,97,98]. Previous rodent studies have found that a bolus dose of 30 µg/kg of TCDD decreased total cholesterol, LDL, and HDL one week post-exposure [99], and that approximately 0.5 mg/kg of PFOA along with a high-fat diet exposure resulted in hypercholesterolemia [65], and approximately 0.26 mg/kg of BPA exposure can also increase total cholesterol [58]. Though the HDL of MIX dams was unchanged, it is unknown how HDL responded at baseline since these data were collected following the glucose tolerance test, and this may reflect acute lipid alterations [100]. Future research is needed to assess lipid profiles in the absence of prior glucose tolerance testing, particularly for HDL and hepatic lipid profiles, to determine the protracted effects of maternal lipid profiles following gestational exposure to EDC mixtures.

There were no significant increases in body volume or visceral adipose volume. MIX dams had an average visceral adipose volume to body volume ratio of 0.09, while vehicle dams had an average bodyweight ratio of 0.07 (*p* = 0.08). Increased visceral adipose is indicative of metabolic syndrome and an increased risk for type 2 diabetes [90]. Adipocytes have been observed to be impacted by single EDC exposures, with BPA found to increase adipogenesis [101] and maternal adipose weight months postpartum following gestational exposure [51]. TCDD was found to upregulate genes in inflammatory pathways in adipose tissue both in vitro and in vivo [102], and numerous other EDCs have been observed to alter the differentiation of adipocytes and expression of adipokines [24,101,102,103]. Furthermore, in epidemiological studies, exposures during pregnancy to phthalates and per and poly-fluoroalkyl substances, such as PFOA, have been associated with elevated long-term maternal weight gain, despite weight gain in pregnancy [23,24]. While such findings are notable, it is possible that a much greater risk is conferred given that women are exposed to multiple EDCs during pregnancy, and the effect of such mixtures on long-term maternal adiposity remains to be investigated [6,7,8,9,31]. Future research should investigate the impact of multiple relevant risk factors, including EDC mixtures, as well as how high-fat diets alter later-life maternal visceral adipose and weight gain in dams exposed during gestation to EDC mixtures and the persistence of such effects.

MIX dams did not show altered serum adipokines: adiponectin [104], insulin [90], monocyte chemoattractant protein-1 (MCP-1) [105], plasminogen activator inhibitor-1 total (PAI-1 Total) [106], or resistin [107], in response to exposures. Fasting and glucose tolerance tests have been shown to alter adipokine levels, which might have led to the lack of adipokine effects seen post-glucose-tolerance test in MIX dams [108,109]. Consequently, future studies are needed to assess adipokine levels at baseline, not after fasting and subsequent glucose-tolerance testing. Though PFOA and TCDD alone resulted in reductions in adiponectin, we did not see a synergistic effect in the MIX. The physiological response from the single EDC exposures might have differed from the MIX exposure, as the MIX-exposed dams might be responding to, or perhaps compensating for, effects from the other EDC exposures, e.g., effects simultaneously of TCDD and PFOA that resulted in a unique metabolic shift or triggered compensation. More work on the combinatorial effects of EDCs is needed to assess these changes. Additionally, circulating steroid hormones, for example, high levels of corticosterone can significantly influence metabolism and blood glucose concentrations by inhibiting energy storage and acting in opposition to insulin [110,111,112]. As such, serum corticosterone levels were assessed following glucose-tolerance testing, but these were not altered by MIX exposure.

It is worth noting, the limitations of the current study include statistical considerations, such as the need for larger sample sizes for identification of effects of single low-dose EDC exposures. If effect sizes of single EDC exposures are small, larger sample sizes are required to confirm effects on these metabolic endpoints. Research comparing low-dose EDC studies should consider the directionality of effects, beta coefficients, and confidence intervals to determine the continuity across replicated studies, even if effects are not statistically significant [113]. Secondly, ANOVA analyses of multiple groups utilize the variance estimates from all groups when determining significance. Therefore, single EDC studies, conducted with just a control and exposed group, would have different variance estimates that may yield different conclusions with respect to statistical significance. Additionally, the single EDC doses utilized were not chosen based on previous effects in pregnant dams. Selecting single EDC doses based on a dose–response curve would have provided more accurate low-doses for the study. Future mixture experiments should utilize dose–response curves, as the doses selected might produce different effects on later-life maternal health. Finally, we targeted the gestational exposure window only. This experiment tests the sensitivity of this window to later-life maternal metabolic reprogramming. However, this is not the reality of human exposures, as women are exposed pre-pregnancy, during pregnancy, and post-pregnancy. Each of these exposure windows may yield differential effects on later-life maternal metabolic health.

Research on maternal metabolic health following gestational EDC exposures should utilize real-world mixtures, along with integrated metabolic assessments, to determine the influence of complex EDC mixtures on women’s health. The current findings indicate that enhanced effects of MIX exposure may result from multiple small hits at different molecular targets that together converge to disrupt the homeostasis of integrated metabolic endpoints, such as serum hyperglycemia and dyslipidemia [87]. Mechanistic studies focused on mixtures should integrate multiple aspects of metabolic physiology, including assessments of the pancreas, liver, and serum lipids, at baseline to understand how such convergence occurs. Future research is needed to advance public health protection of women by assessing the influence of EDC mixtures on later-life maternal metabolic health to ensure “safe” exposure levels are not overestimated.

## 5. Conclusions

In conclusion, exposure during pregnancy to a curated mixture of EDCs, known to target metabolism but not exposures to single EDCs, altered maternal glucose tolerance, consistent with protracted toxicity and potential reprogramming. Moreover, this protracted alteration of blood glucose levels following MIX was unique to pregnancy as non-pregnant female mice showed no effects. These data indicate pregnancy is a unique window of EDC exposure and support the possibility that mixtures of low-dose EDCs may influence later-life maternal metabolic disease risk. More work is needed with larger sample sizes and more research is needed focused on grouping EDCs for mixture risk assessment based on common adverse health outcomes.

## Figures and Tables

**Table 1:** EDC Dosing Method.

EDC	Dose	Solvent	Dose Reference
Atrazine (ATR)	10 mg/kg	Peanut Oil	Below levels typically shown to cause effects in animal studies [73,74,75,76]
Bisphenol-A (BPA)	50 μg/kg	Peanut Oil	Current oral human daily reference dose (https://www.epa.gov/iris; accessed on 1 September 2021)
Perfluorooctanoic acid (PFOA)	0.10 mg/kg	Distilled water	At or below NOAEL referenced in animal studies in the EPA 2016 document ‘Health Effects Support Document for Perfluorooctanoic Acid (PFOA)’ [80] Below levels typically shown to cause effects in animal studies [65,77]
2,3,7,8-tetrachlorodibenzo-p-dioxin (TCDD)	0.036 μg/kg	Anisole diluted in peanut oil	Below levels typically shown to cause effects in animal studies [69,70,72,79]
Mixture (MIX)	All the above doses added together	All the above	All the above

## Data Availability

The data presented in this study are available on request from the corresponding author.
